# A novel gene network inference algorithm using predictive minimum description length approach

**DOI:** 10.1186/1752-0509-4-S1-S7

**Published:** 2010-05-28

**Authors:** Vijender Chaitankar, Preetam Ghosh, Edward J Perkins, Ping Gong, Youping Deng, Chaoyang Zhang

**Affiliations:** 1 School of Computing, University of Southern Mississippi, MS 39402, USA; 2 Environmental Laboratory, U.S. Army Engineer Research and Development Center, 3909 Halls Ferry Road, Vicksburg, MS 39180, USA; 3 SpecPro Inc., 3909 Halls Ferry Road, Vicksburg, MS 39180, USA

## Abstract

**Abstract:**

**Background**

Reverse engineering of gene regulatory networks using information theory models has received much attention due to its simplicity, low computational cost, and capability of inferring large networks. One of the major problems with information theory models is to determine the threshold which defines the regulatory relationships between genes. The minimum description length (MDL) principle has been implemented to overcome this problem. The description length of the MDL principle is the sum of model length and data encoding length. A user-specified fine tuning parameter is used as control mechanism between model and data encoding, but it is difficult to find the optimal parameter. In this work, we proposed a new inference algorithm which incorporated mutual information (MI), conditional mutual information (CMI) and predictive minimum description length (PMDL) principle to infer gene regulatory networks from DNA microarray data. In this algorithm, the information theoretic quantities MI and CMI determine the regulatory relationships between genes and the PMDL principle method attempts to determine the best MI threshold without the need of a user-specified fine tuning parameter.

**Results**

The performance of the proposed algorithm was evaluated using both synthetic time series data sets and a biological time series data set for the yeast *Saccharomyces cerevisiae*. The benchmark quantities precision and recall were used as performance measures. The results show that the proposed algorithm produced less false edges and significantly improved the precision, as compared to the existing algorithm. For further analysis the performance of the algorithms was observed over different sizes of data.

**Conclusions**

We have proposed a new algorithm that implements the PMDL principle for inferring gene regulatory networks from time series DNA microarray data that eliminates the need of a fine tuning parameter. The evaluation results obtained from both synthetic and actual biological data sets show that the PMDL principle is effective in determining the MI threshold and the developed algorithm improves precision of gene regulatory network inference. Based on the sensitivity analysis of all tested cases, an optimal CMI threshold value has been identified. Finally it was observed that the performance of the algorithms saturates at a certain threshold of data size.

## Background 

A gene regulatory network represents regulatory interactions between genes that can be established from measuring how the expression level of one affects the expression level of the others [1]. DNA microarray experiments provide expression levels of thousands of genes under different conditions. A DNA microarray dataset is generally in the form of a matrix where rows correspond to genes and columns correspond to conditions, or vice versa. Reverse engineering is the process of finding the regulatory relationships between genes based on DNA microarray data. Reverse engineering of gene regulatory networks remains a major issue and area of interest in the field of bioinformatics and systems biology. According to a recent review paper [2], there have been a number of models related to this area *viz.* Bayesian Networks [3], Dynamic Bayesian Networks [4], Boolean Networks [5,6], Probabilistic Boolean Networks [7,8], Differential Equation Models [9] and Information Theory Models [10-14].

This study deals with reverse engineering of gene regulatory networks from DNA microarray data, where no gold standard method exists. Each method has its own advantages and disadvantages. Based on simulations of different models it has been observed that differential equation models and dynamic Bayesian networks have high accuracy but they are computationally expensive and hence are applicable to only small datasets. Boolean networks can be used to study coarse grained properties of genetic networks but it requires the data to be quantized to 0 or 1. Thus, these models cannot be used to study fine grained properties. Also, the main limitation of Boolean networks is their inherent determinism [7], which can be solved by using probabilistic Boolean networks. However, they still cannot be used to study fine grained properties of genetic networks. Information theoretic models gained much attention due to their simplicity and low computational costs. Because of their low data requirements, they are suitable to infer even large-scale networks. Thus, they can be used to study global properties of large-scale regulatory systems [2].

### Related work

A number of algorithms that implement information theoretic approaches have been proposed in the past [10-14]. The regulatory relationships between genes are derived based on MI in all these algorithms. MI measures the amount of information that can be obtained about one random variable by observing another one. Compared with the correlation coefficient based metric, the MI is suitable for nonlinear relations and represents a good metric for evaluating the dependency between two random variables [15].

The following assumptions were made in the past 

1.  If the MI value is low, then genes are not connected

2.  If the MI value is high, then genes are connected. 

Based on the study of chemical kinetics, it has been found that the second assumption is not true [11]. If there are two genes being regulated by a third gene, then the MI between the two genes could be high resulting in a false edge in the network. ARACNE [13] is the first inference algorithm to implement a method to identify such false edges. ARACNE states that if the MI between two genes X and Y is less than or equal to that between genes X and Z or between Y and Z, i.e. I(X, Y) ≤ [I(X, Z), I(Y, Z)], then there is no connectivity between X and Y.  The ARACNE method loses validity in other cases like Z → X, Y [11]. To deal with such cases, Zhao et al. [11] exploited the concept of CMI, and was the first to implement both MI and CMI to infer gene regulatory networks from DNA microarray data. However, selecting a MI or CMI threshold is the major drawback in their approach.

The MDL principle [16-19] has been implemented in [10,12] to estimate the best MI threshold.  Various implementations of the MDL principle have been studied extensively in [18,19]. The algorithm proposed in [10] often yields good results, but it does so with an *ad hoc* coding scheme that requires a user-specified tuning parameter. Dougherty et al. [12] implemented the normalized maximum likelihood model to overcome this issue. In our proposed algorithm, we implement the PMDL principle which is well suited for time series data and combine it with the CMI metric. In particular, our scheme requires only one threshold parameter as against two threshold values that need to be specified in the scheme proposed in [11]. There exist a number of information theoretic approaches to inferring gene regulatory networks. They rely on threshold values and/or fine tuning parameters. One approach [12] does not involve any fine tuning parameters or threshold values but it does not utilize CMI which is useful in reducing false edges in a network. Our goal is to develop an algorithm which reduces the number of fine tuning parameters and thresholds and improve the performance of algorithm using higher order information theoretic quantities such as CMI.

### Contributions

1.    Our major contribution is the implementation of PMDL principle that eliminates the need of a fine tuning parameter. 

2.    Our work combines the PMDL principle with CMI for the first time to achieve better performance.

3.    CMI has been used in the past but our scheme adds directions derived from an ad hoc time delay. 

4.    We report the threshold sensitivity of gene regulatory network inference schemes for the first time as it gives the users an estimate of the range of thresholds which should be used.

5.    We report for the first time the effect of the size of DNA microarray data.

A preliminary version of this work appeared in [20].

## Results and discussion

### Simulation on random synthetic networks

The proposed algorithm is compared with [10] on synthetic random networks. The algorithm proposed in [10] by Zhao et al. is also referred as Network MDL in this paper. Benchmark measures like recall (*R*) and precision (*P*) are used to evaluate the performance of inference algorithms. While different definitions exist [21], *R* is herein defined as *C_e_*/(*C_e_*+*M_e_*) and *P* as *C_e_*/(*C_e_*+*F_e_*), where *C_e_* denotes the edges that exist in both the true and the inferred network, *M_e_* are the edges that exist in the true network but not in the inferred network and *F_e_* are the edges that do not exist in the true network but do exist in the inferred network.

**Figure 1:**
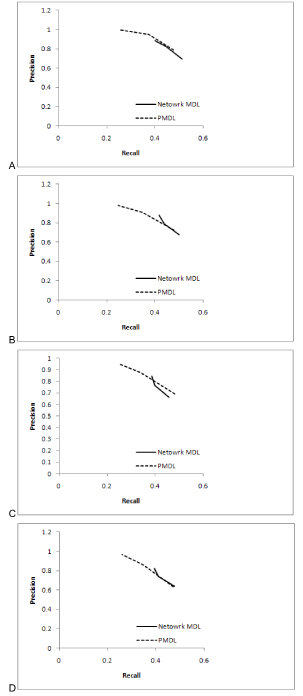
**Precision vs. recall curves for (A) 20 genes (B) 30 genes (C) 40 genes (D) 50 genes. The data is quantized to 2 levels in all cases.** The precision vs. recall graphs for the network MDL and PMDL algorithms are shown in this figure.

For a specific size of the network, both the algorithms are run for different threshold values 30 times each and the average of *P* and *R* are calculated. The algorithms are run for 20, 30, 40 and 50 numbers of genes. The *P* vs. *R* curves for each of these networks with different threshold values are given in Figure 1.

Zhao et al. [10] reported 0.2 to 0.4 as suitable values for the tuning parameter, hence we use the values 0.2, 0.3 and 0.4 to build the networks. Based on simulations of the proposed algorithm, we found that the threshold for CMI worked best for values in the range 0.1 to 0.2. Thus, threshold values 0.1, 0.15 and 0.2 were used to build the networks.

In Figure 1 it is observed that the *P* of the proposed algorithm is higher but *R* is lower in most of the cases as compared to network MDL. The number of false negatives is fewer in the proposed algorithm and as most biologists are interested in true positives, our proposed algorithm is preferred over the network MDL.

### Performance on *Saccharomyces cerevisiae* data set

The time series DNA microarray data from [22] was used to infer gene regulatory networks. The Spellman experiment was chosen because it provides a comprehensive series of gene expression datasets for Yeast cell cycle. In the experiment four time series expression datasets were generated using four different cell synchronization methods: cdc15, cdc28, alpha-factor and elutriation with 24, 17, 18 and 14 time points respectively. The alpha-factor dataset contained more time points than the cdc28 and the elutriation dataset with fewer missing values than the cdc15 dataset. Therefore, we chose to use the alpha-factor dataset to infer gene regulatory networks.

As mentioned earlier, pre-processing plays an important part in reverse engineering process. As there were some missing values in the data we pre-processing the data as in [10]. Initially the data was quantized to 0 or 1. In order to quantize the expression values of every gene they were sorted in ascending order and the first and last values of the sorted list were discarded as outliers, then the upper 50% are converted to 1 and the lower 50% is converted to 0. Any missing time points are set to the mean of their respective neighbours [10]. If the missing time point is the first or the last one it is set to the nearest time point value.

**Figure 2:**
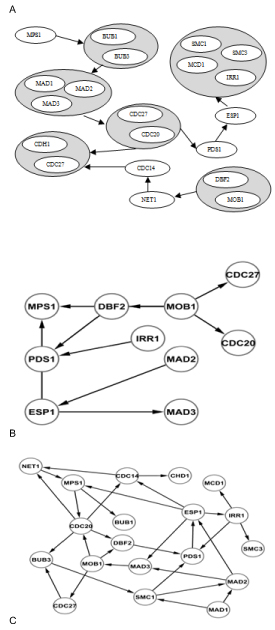
**Networks for (A) Biological network (B) Network MDL approach (C) PMDL approach** The performance on the algorithms is performed on a biological data set. The biological network is derived from the yeast cell cycle pathway in KEGG database.

The true biological network used for comparison purposes was derived from the yeast cell cycle pathway [23-25]. A total of 6 networks were reverse engineered, of which three were inferred using our proposed method and the remaining three using [10]. The best network out of the three in each case was used for comparison. The true network and the networks reverse engineered using the proposed algorithm and the network MDL are shown in Figure 2. Of the 30 edges inferred by our approach nine are correctly inferred edges. The method proposed in [10] inferred a total of nine edges, of which only one is correctly inferred edge. The results favour our approach. 

### Threshold sensitivity

Here we report the performance of our scheme based on different values of the user specified threshold over synthetic networks. For threshold values of 0.15 and 0.2, a high precision (over 90% in most cases) was observed but the recall for these thresholds was low (from 25% to 30%) as compared to a threshold value of 0.1 which had a fair recall (over 47%) and good precision (63% to 79%) performance.

**Figure 3:**
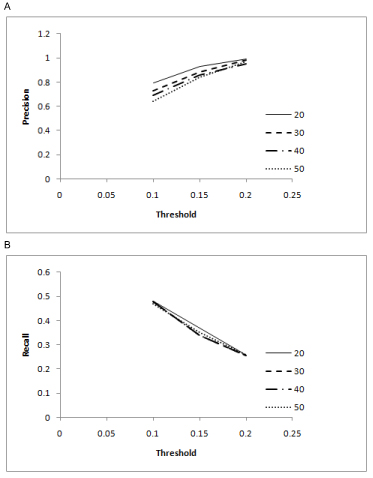
**(A) Precision Sensitivity (B) Recall Sensitivity** In this figure we report the performance of our scheme based on different values of the user specified threshold (Conditional Mutual Information).

Figure 3 indicates that as the threshold value is increased, precision increases while the recall decreases. The simulation experiments show that 0.1 is the optimal threshold value.

### Time and space complexities

The performance of the algorithm depends on three factors. The number of genes, the number of time points and most importantly the number of parents inferred for each gene by the algorithm. To see what role these factors play we looked into the time and space complexities of the algorithm.

Step 4 of the algorithm iterates *n*^2^*m* times, where n is number of genes and *m* is the number of time points; from line 5 to line 18 the algorithm iterates *n*^4^ times; lines 15 and 16 of the algorithm iterates *n*^3^*m* times. Finally, from lines 20 to 31 the algorithm iterates *n*^3^ times. Thus, the time complexity of the algorithm is Θ(*n*^4^ + *n*^3^*m*).

From the time complexity it can be seen that if the number of genes is larger than the number of time points then the run time depends more on the number of genes. And if the number of time points is larger than the number of genes then the run time depends more on the number of time points.

When it comes to space complexity the conditional probability tables play a major role. If a gene has *n* parents then the conditional probability tables take 2*^n^* units of space. Thus, the amount of memory needed by the algorithm depends on the number of parents inferred by the network. As the space complexity grows exponentially based on the number of parents it is possible that the algorithm may run out of memory for a data set with as few as 50 genes but run for as little as 5 minutes for a data set with several hundred genes. There are 2 ways to overcome this limitation: 

1. Restrict the number of parents and

2. Take the next smallest description length, instead of using the smallest one.

The first approach guarantees results when the number of parents is restricted to small values but this may lower the accuracy of the result. The second approach may take more time to run but as we are not restricting the number of parents the accuracy of the algorithm is not affected. We plan to perform some bench marking studies on the above two approaches to see which one works better.

### Data requirements:

**Figure 4:**
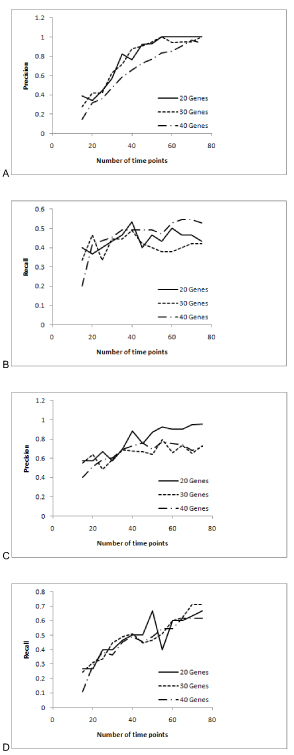
**Precision and Recall Graphs for PMDL and Network MDL.** This figure reports the performance of the algorithm over different size of data.

A network and a data set with 75 time points were generated. Each of the algorithms was run 13 times starting with the first 15 time points. An increment of 5 time points was made for every subsequent run. For every run the values of precision and recall were computed. In Network MDL the free parameter was set to 0.2 and in PMDL algorithm the conditional mutual information threshold was set to 0.1. The plots for precision and recall are as shown in Figure 4.

For the PMDL algorithm it is observed from Figure 4 that the precision increased till 55 time points and beyond that the precision decreased or increased for one network. For another network again precision increased till 55 time points and then saturated.  For another network the precision increased till 70 time points and then decreased at final 75 time points. The recall for PMDL algorithm increased till 40 time points and beyond that the recall increased or decreased. For Network MDL algorithm it was observed that precision increased till 35 time points and beyond that the recall increased or decreased. The recall for Network MDL algorithm increased till 40 time points and beyond that the recall increased or decreased.

**Figure 5:**
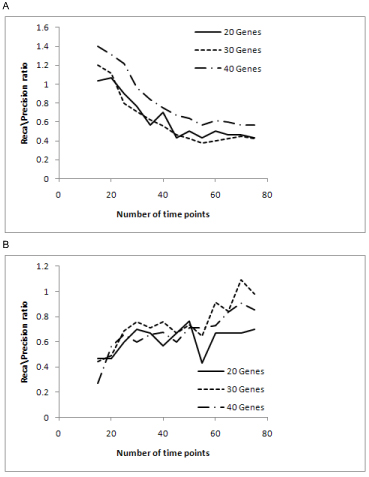
**Recall/Precision ratio graphs for (A) PMDL and (B) Network MDL.** The graphs recall/precision ratio for both PMDL and network MDL are shown here.

**Figure 6:**
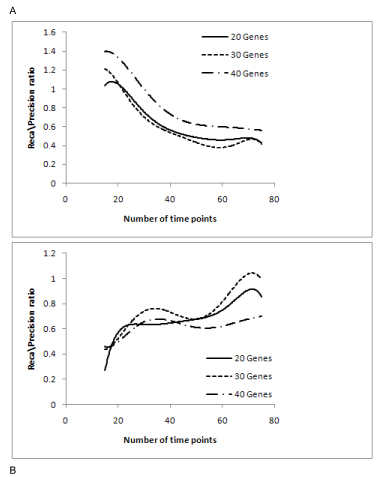
**Recall/Precision ratio curve fit graphs for (A) PMDL and (B) Network MDL.** The recall/precision ratio curve fits for the algorithms are shown in this figure

For further analysis we considered the recall/precision ratio (Figure 5). Saturation beyond 55 time points was observed for PMDL for recall/precision ratio. Saturation was also found from 30 to 45 time points for Network MDL algorithm and beyond that the ratio either increased or decreased. The curve fits of Figure 5 are shown in Figure 6.

## Conclusions 

We have proposed a new gene regulatory inference algorithm that implements the PMDL principle. The simulation results show that the PMDL principle is fair in determining the MI threshold. A problem with the proposed algorithm is determining the CMI threshold. We have tested the sensitivity of the threshold and based on the performance of our scheme we identified that the value of 0.1 is optimal for most synthetic networks. This was also true in the case of reverse engineering of gene regulatory networks from biological time series DNA microarray data. In synthetic network simulations the proposed algorithm produced fewer false edges compared to [10]; however, it resulted in a larger number of missing edges.  We plan to improve this issue of our algorithm in the future. Currently the space complexity of the algorithm increases exponentially based on the number of parents inferred for genes. We also plan to improve this in the future. Finally we studied the effects of different data sizes over the algorithms. It was observed that the performance of PMDL saturates after 50 time points, saturation for network MDL was observed between 30 to 45 time points and beyond 45 time points the performance increased or decreased (better than saturation point). We plan to study the effects of data sets with more than 75 time points in the future.

## System and methods

### Genetic network

The network formulation is similar to the one used in [10]. A graph *G*(*V, E*) represents a network where *V* denotes a set of genes and *E* denotes a set of regulatory relationships between genes. If gene *x* shares a regulatory relationship with gene *y*, then there exists an edge between *x* and *y* (*x* → *y*). Genes can have more than one regulator. The notation *P*(*x*) is used to represent a set of genes that share regulatory relationships with gene *x.* For example, if gene  shares a regulatory relationship with *y* and *z* then *P*(*x*) = {*y, z*}. Also every gene is associated with a function *f_x_P*(*x*) which denotes the expression value of gene *x* determined by the values of genes in *P*(*x*). 

The gene expression is affected by many environmental factors. Since it is not possible to incorporate all factors the regulatory functions are assumed to be probabilistic. Also, the gene expression values are assumed discrete-valued and the probabilistic regulation functions are represented as look-up tables. If the expression levels are quantized to *q* levels and a gene *x* has *n* predecessors then the look up table has *q^n^* rows and *q* columns and every entry in the table corresponds to a conditional probability.

Say we have a gene *x* which shares regulatory relationship with two other genes *y,z* and the data is quantized to 2 levels, the look up table is as in Table 1. In this example the entry 0.6 can be inferred as, if genes *y* and *z* are lowly expressed then the probability that *x* is also lowly expressed is 0.6.

**Table 1:** Conditional probability table

*yz:x*	0	1
00	0.6	0.4
01	0.3	0.7
10	0.5	0.5
11	0.8	0.2

Given a gene with two parents the table shows an example conditional probability table.

### Information theoretic quantities

***Entropy:*** Entropy (H) is the measure of average uncertainty in a random variable. Entropy of a random variable *X* with probability mass function *p*(*x*) is defined [15] by

	(1)

***Mutual information:*** MI measures the amount of information that can be obtained about one random variable by observing another one. Since MI by itself does not contain directional information, using ad hoc time delay has been proposed in the past to overcome this issue. The gene system is assumed to be event driven, i.e. all the regulations are performed step by step and in each step all regulations happen only once. Therefore, the latency parameter is set by default to a unit step.

MI is defined [15] as

	(2)

MI can also be defined in terms of entropies as 

*I*(*X*;*Y*) = *H*(*X*) + *H*(*Y*) − *H*(*X*, *Y*)	(3)

As stated earlier this above quantity does not contain directional information and hence a time lag is introduced and the quantity after time lag is estimated as:

*I*(*X_t_*;*Y*_*t*+1_) = *H*(*X_t_*) + *H*(*Y*_*t*+1_) − *H*(*X_t_*,*Y*_*t*+1_)	(4)

**Figure 7:**
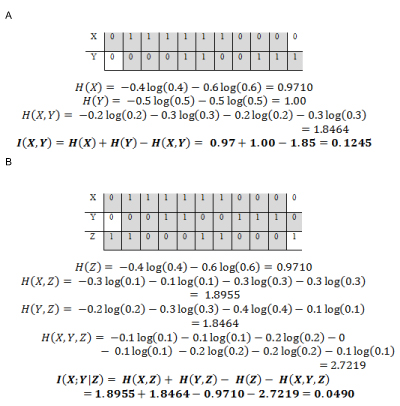
**Example calculations for (A) MI and (B) CMI.** This figure gives examples to calculate the basic Information Theory quantities: Mutual Information and Conditional Mutual Information.

Figure 7 (A) shows an example for calculating mutual information between random variables.

***Conditional mutual information:*** High MI indicates that there may be a direct or indirect relationship between the genes. CMI is the reduction in the uncertainty of *X* due to knowledge of *Y* when *Z* is given [15]. Time lag between variables is considered to give a sense of direction. The CMI of random variables *X* and *Y* given *Z* is defined [15] as

	(5)

CMI can also be expressed in terms of entropies as:

*I*(*X*; *Y*|*Z*) = *H*(*X*, *Z*) + *H*(*Y*, *Z*) − *H*(*Z*) − *H*(*X*, *Y*, *Z*)	(6)

Again this quantity does not contain directional information. After introducing time lag the quantity is estimated as:

*I*(*X_t_*;*Y*_*t*+1_|*Z_t_*) = *H*(*X_t_*, *Z_t_*) + *H*(*Y*_*t*+1_, *Z_t_*) − *H*(*Z_t_*) − *H*(*X_t_*, *Y*_*t*+1_, *Z_t_*)	(7)

Figure 7 (B) shows an example for calculating conditional mutual information between random variables.

### Entropy calculations

The proposed algorithm deals with quantized data. In general, it is assumed that the q-level quantization admits the alphabet *A_q_* = {0, 1, …, *q* − 1} then, the probability mass function from *m* amples *s*_1_, …, *s_m_* is estimated as

	(8)

Where, 1_{.}_(.) is the indicator function, defined as

	(9)

By substituting (9) in (1) entropies can be estimated and these entropy estimates can be substituted in (3) and (6) to obtain the MI and CMI estimates [11].

### Predictive minimum description length principle

The description length of the two-part MDL principle involves calculation of the model length and the data length. As the length can vary for various models, the method is in danger of being biased towards the length of the model [19]. The Normalized Maximum Likelihood Model has been implemented in [12] to overcome this issue. Another such model based on universal code length is the PMDL principle.

We chose to implement the PMDL principle as it suits time series data [17].  The concept of PMDL principle model was proposed in [26,27].

The description length for a model in PMDL [17,26] is given as 

Where *p*(*X*_*t*+1_|*X_t_*) is the conditional probability or density function. We calculate the description length as data length given in [10].

A gene can take any value when transformed from one time point to another due to the probabilistic nature of the network. The network is associated with Markov chain which is used to model state transitions. These states are represented as n-gene expression vectors *X_t_* = (*x*_1,*t*_, …, *x_n,t_*)*^T^* and the transition probability *p*(*X*_*t*+1_|*X_t_*) can be derived as follows:

	(10)

The probability *p*(*x*_*i,t*+1_| ℙ*_t_*(*x_i_*) can be obtained from the look-up table associated with the vertex *x_i_* and is assumed to be time invariant. It is estimated as follows:

	(11)

Each state transition brings new information that is measured by the conditional entropy:   

*H*(*X*_*t*+1_|*X_t_*)= − log(*p*(*X*_*t*+1_|*X_t_*))	(12)

The total entropy for given m time-series sample points, (*X*_1_, …, *X_m_*) is given by

	(13)

As *H*(*X*_1_) is same for all models it is removed thus the description length is 

	(14)

### Inference algorithm

**Figure 8:**
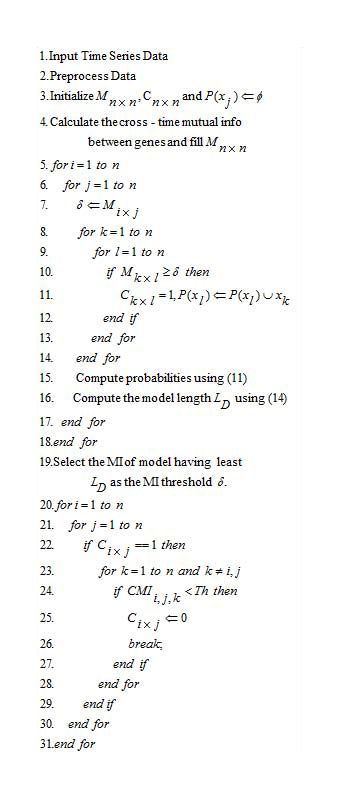
**Inference algorithm** This figure shows the Predictive Minimum Description Length Principle inference algorithm.

Given the time series data, the data was first pre-processed, which involved filling missing values and quantizing the data. Then the MI matrix *M*_*n*×*n*_ was evaluated using (4). A connectivity matrix *C*_*n*×*n*_ was maintained which had two entries: 0 and 1. An entry of 0 indicates that no regulatory relationship exists between genes, but an entry of 1 at *C*_*i*×*j*_ indicates that gene *i* regulates *j*. The algorithm is given in Figure 8. From lines 5 to 18 every value of the MI matrix is used as a threshold and a model is obtained. The conditional probabilities and the description lengths for each of these models are evaluated using (11) and (14) respectively. Then at line 19 the MI which was used to obtain the model with the shortest description length is then used as the MI threshold (δ) to obtain the initial connectivity matrix. From lines 20 to 31, for every valid regulatory connection in the connectivity matrix, the CMI of the genes with every other gene is evaluated using (7) and if the value is below the user specified threshold (*Th*) the connection is deleted.

## Competing interests

The authors declare that they have no competing interests.

## Authors' contributions

VC, CZ and PG1 developed the algorithm and implemented the algorithm on synthetic and biological data sets. An in-depth analysis of results was also performed on the results. EP, PG2 and YD coordinated the study. All authors read and approved the final manuscript.
